# Analysis of the Factors Contributing to Bariatric Success After Laparoscopic Redo Bariatric Procedures: Results from Multicenter Polish Revision Obesity Surgery Study (PROSS)

**DOI:** 10.1007/s11695-022-06306-3

**Published:** 2022-10-15

**Authors:** Michał Łabul, Michał Wysocki, Katarzyna Bartosiak, Michał Orłowski, Bartosz Katkowski, Paweł Jaworski, Piotr Małczak, Piotr Major, PROSS–Collaborative Study Group, Piotr Major, Michał Pędziwiatr, Justyna Rymarowicz, Piotr Zarzycki, Tomasz Stefura, Karol Ciszek, Piotr Małczak, Piotr Myśliwiec, Hady Razak Hady, Paulina Głuszyńska, Monika Proczko-Stepaniak, Michał Szymański, Maciej Walędziak, Michał Janik, Andrzej Kwiatkowski, Magdalena Materlak, Katarzyna Bartosiak, Łukasz Czyżykowski, Maciej Mawlichanów, Piotr Kowalewski, Jacek Szeliga, Wojciech Kupczyk, Natalia Dowgiałło-Gornowicz, Paweł Lech, Anna Harań, Grzegorz Kowalski, Rafał Mulek, Michał Krefft, Michał Wysocki, Michał Orłowski, Paula Franczak, Artur Binda, Wiesław Tarnowski, Paweł Jaworski, Mateusz Kamiński, Maciej Pastuszka, Wojciech Lisik, Paweł Szymański, Bartosz Katkowski, Michał Leśniak, Michał Łabul

**Affiliations:** 1 Department of General Surgery, Specialist Hospital in Legnica, Legnica, Poland; 2 Department of General Surgery and Surgical Oncology, Ludwik Rydygier Memorial Hospital, Cracow, Poland; 3 Department of General, Oncological, Metabolic and Thoracic Surgery, Military Institute of Medicine, Warsaw, Poland; 4 Department of General and Oncological Surgery, Ceynowa Hospital, Wejherowo, Poland; 5 Department of General and Vascular Surgery, Polanica Zdrój, Poland; 6 Department of General, Oncological and Digestive Tract Surgery, Centre of Postgraduate Medical Education, Orłowski Hospital, Warsaw, Poland; 7 2nd Department of General Surgery, Jagiellonian University Medical College, Cracow, Poland

**Keywords:** Redo bariatric procedure, Revisional obesity surgery, Weight regain, Insufficient weight loss, Obesity-associated diseases, Success factor

## Abstract

**Introduction:**

With continuously growing number of redo bariatric surgeries (RBS), it is necessary to look for factors determining success of redo-surgeries.

**Patients and methods:**

A retrospective cohort study analyzed consecutive patients who underwent RBS in 12 referral bariatric centers in Poland from 2010 to 2020. The study included 529 patients. The efficacy endpoints were percentage of excessive weight loss (%EWL) and remission of hypertension (HT) and/or type 2 diabetes (T2D).

**Results:**

Group 1: weight regain

Two hundred thirty-eight of 352 patients (67.6%) exceeded 50% EWL after RBS. The difference in body mass index (BMI) pre-RBS and lowest after primary procedure < 10.6 kg/m2 (OR 2.33, 95% CI: 1.43–3.80, *p* = 0.001) was independent factor contributing to bariatric success after RBS, i.e., > 50% EWL.

Group 2: insufficient weight loss

One hundred thirty of 177 patients (73.4%) exceeded 50% EWL after RBS. The difference in BMI pre-RBS and lowest after primary procedure (OR 0.76, 95% CI: 0.64–0.89, *p* = 0.001) was independent factors lowering odds for bariatric success.

Group 3: insufficient control of obesity-related diseases

Forty-three of 87 patients (49.4%) achieved remission of hypertension and/or type 2 diabetes. One Anastomosis Gastric Bypass (OAGB) as RBS was independent factor contributing to bariatric success (OR 7.23, 95% CI: 1.67–31.33, *p* = 0.008), i.e., complete remission of HT and/or T2D.

**Conclusions:**

RBS is an effective method of treatment for obesity-related morbidity. Greater weight regain before RBS was minimizing odds for bariatric success in patients operated due to weight regain or insufficient weight loss. OAGB was associated with greater chance of complete remission of hypertension and/or diabetes.

**Graphical abstract:**

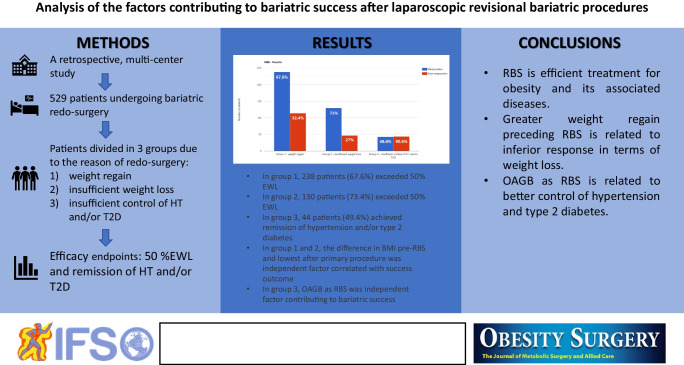

**Supplementary Information:**

The online version contains supplementary material available at 10.1007/s11695-022-06306-3.

## Introduction

Bariatric surgery is effective and approved method of treatment of clinically severe obesity, however, we must recognize its limitations and realize that some patients, due to various reasons, will require redo bariatric surgeries. Majority of bariatric patients manages to achieve satisfactory weight loss, i.e., > 50% EWL, but approximately 15–20% do not achieve or sustain this goal [1]. Inadequate body weight loss, weight regain, and unsatisfactory control of associated medical *conditions* are the most common causes for redo bariatric surgeries (RBS) [2]. More indications for RBS include gastroesophageal reflux disease (GERD) and other complications of primary bariatric surgery (PBS) such as marginal ulcers, malnourishment, and fistulas [3]. Prevalence of GERD increases after sleeve gastrectomy and with vast popularity of this procedure poses a serious problem for patients and surgeons in quality of life, but also as possible risk factor for Barrett’s esophagus and later on esophageal cancer [4]. RBS represent around 7% of the total bariatric procedures in the world [5]. In some countries, including the USA, RBS are nowadays the third most common bariatric surgeries in total [6]. As the number of bariatric surgeries performed continuously increases worldwide [7–9], it is safe to assume that demand for RBS will also grow.

Indications for RBS are not clearly defined. Moreover, these procedures are technically more challenging and associated with higher morbidity rate [10]; thus, patients that are considered for RBS require individual evaluation of potential benefits and risks. In order to improve the outcomes of treatment and avoid inaccurate qualifications, it is important to learn about factors influencing the effectiveness of RBS.

## Purpose

The aim of this study was to investigate predictive factors for achieving bariatric success in terms of postoperative weight loss and remission of obesity-related diseases 1 year after redo bariatric procedures.

## Patients and Methods

### Methods

A retrospective cohort study analyzed consecutive patients who underwent redo surgical treatment for clinically severe obesity in 12 referral bariatric centers in Poland from January 2010 to January 2020. Inclusion criteria are redo bariatric surgery after prior surgical treatment of obesity, laparoscopic approach, and patients ≥ 18 years and older. Bariatric operation performed after intragastric balloon treatment was not considered as a redo bariatric surgery. The exclusion criteria were RBS due to peri- or postoperative morbidity of primary procedure, the inability to collect necessary data, and incomplete 12 months bariatric follow-up after RBS. Each of participating bariatric center provided specific data, which were processed and used in the overall analysis. The study population was divided into three groups: group 1, patients with weight regain after primary procedure; group 2, patients with insufficient weight loss after primary procedure; and group 3, patients with insufficient control of obesity-associated diseases after primary procedure.

Patients who had RBS performed as a consequence of a long-term complications such as GERD, band-associated issues, malnourishment, and persistent vomiting are heterogenous group, and as such setting, a clear definition of success of RBS in those patients is very difficult. Because of that, we decided to exclude this group from this study.

In groups 1 and 2, bariatric success was defined as achieving over 50% EWL (excess weight loss) at the end of observation period. %EWL was calculated as following: (maximal lifetime weight – post-RBS weight)/(maximal lifetime weight – ideal weight), in which ideal weight was assumed as for the BMI = 22. In group 3, bariatric success was defined as a complete remission of hypertension and/or diabetes after RBS. Diabetes remission criteria was set according to ADA (American Diabetes Association) guidelines, which is defined as glycated hemoglobin (HbA1c) levels below 48 mmol/mol (6.5%) achieved at least 3 months after withdrawal of glucose-lowering medications [11]. Perioperative morbidity was defined as any deviation from the standard perioperative course after RBS that required additional measures to correct within 30 days after procedure. Postoperative morbidity of RBS was defined as morbidity in the first 12 months after procedure, including perioperative morbidity. Each patient qualified for surgical treatment in accordance with The Polish Guidelines for Metabolic and Bariatric Surgery [12]. The length of biliopancreatic limb in OAGB was estimated intraoperatively to be 180–250 cm, depending on patient’s BMI, age, and diabetes status, following the rule by Garcia-Caballero et al. [13]. The length of left common limb was estimated to be 350–400 cm. In RYGB, the length of biliopancreatic limb was estimated to be 100 cm and the length of alimentary limb to be 150 cm. All the surgical procedures were performed laparoscopically, and the perioperative care was based on standardized protocols, which ensured reliable data comparison [14, 15].

### Statistical Analysis

Statistica 13.3 PL software (Tibco, CA, USA) was used for statistical analyses. Continuous values were presented as means with standard deviations or medians with interquartile ranges when appropriate. Qualitative variables were compared using the Pearson χ-square with or without Yates’ correction. Significant variables in univariate logistic regression models were then adjusted in multivariate analysis to obtain significant, independent risk factors and to calculate the OR with 95% confidence interval (CI). *P* values ≤ 0.05 were considered statistically significant.

### Patients

The study included 529 patients, including 134 men (25.3%) and 395 women (74.7%). The median age of patients who underwent redo bariatric surgery was 43 (37–51) years. Group 1 consisted of 352 patients, group 2 of 177 patients, while group 3 of 87 patients. All patients from group 3 also were included in group 1 (*n* = 52) or group 2 (*n* = 35).

**Fig. 1:**
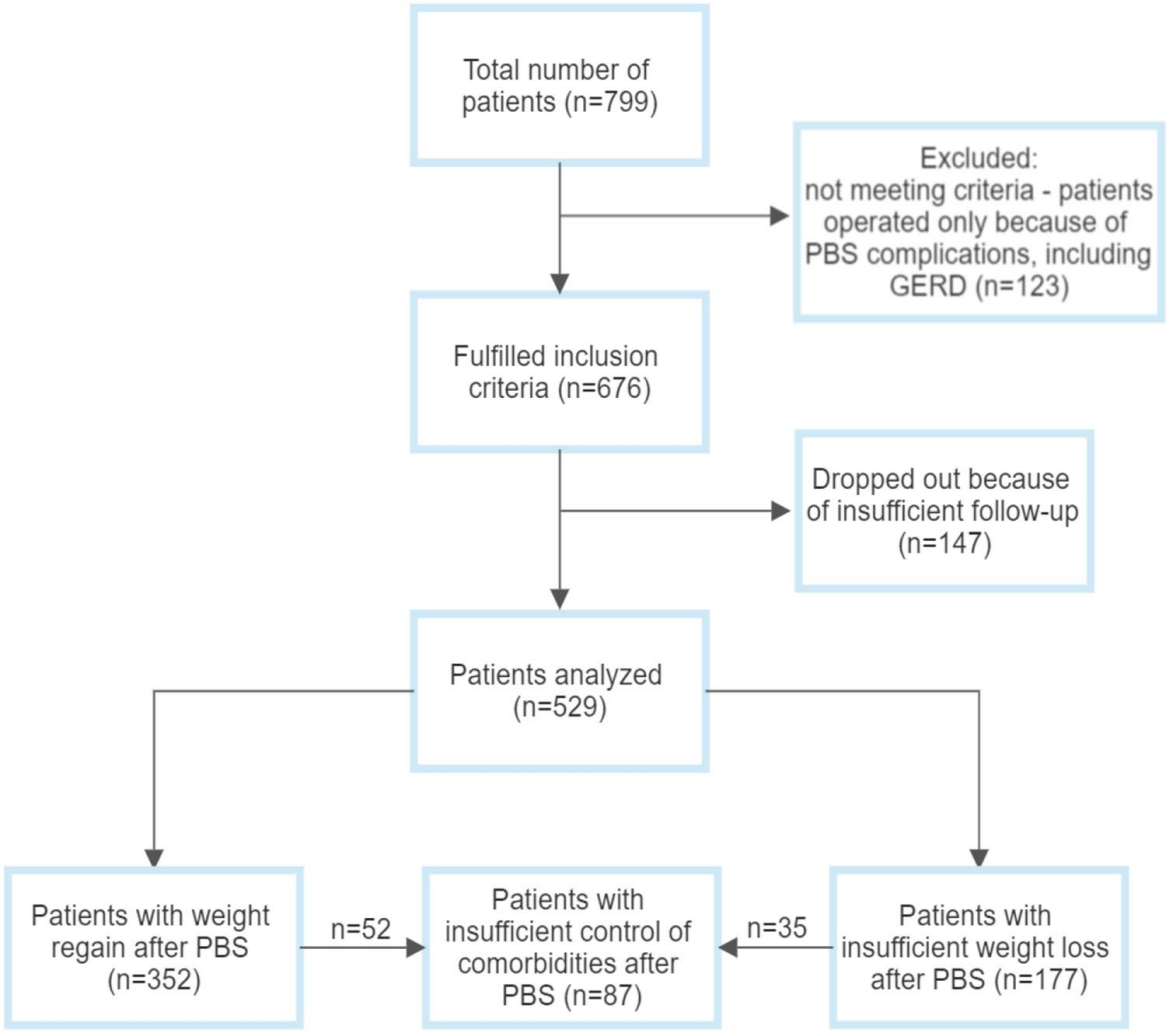
Flowchart of patients in the study

Flow chart of the patients included in the study is presented in Fig. 1.

## Results

**Fig. 2:**
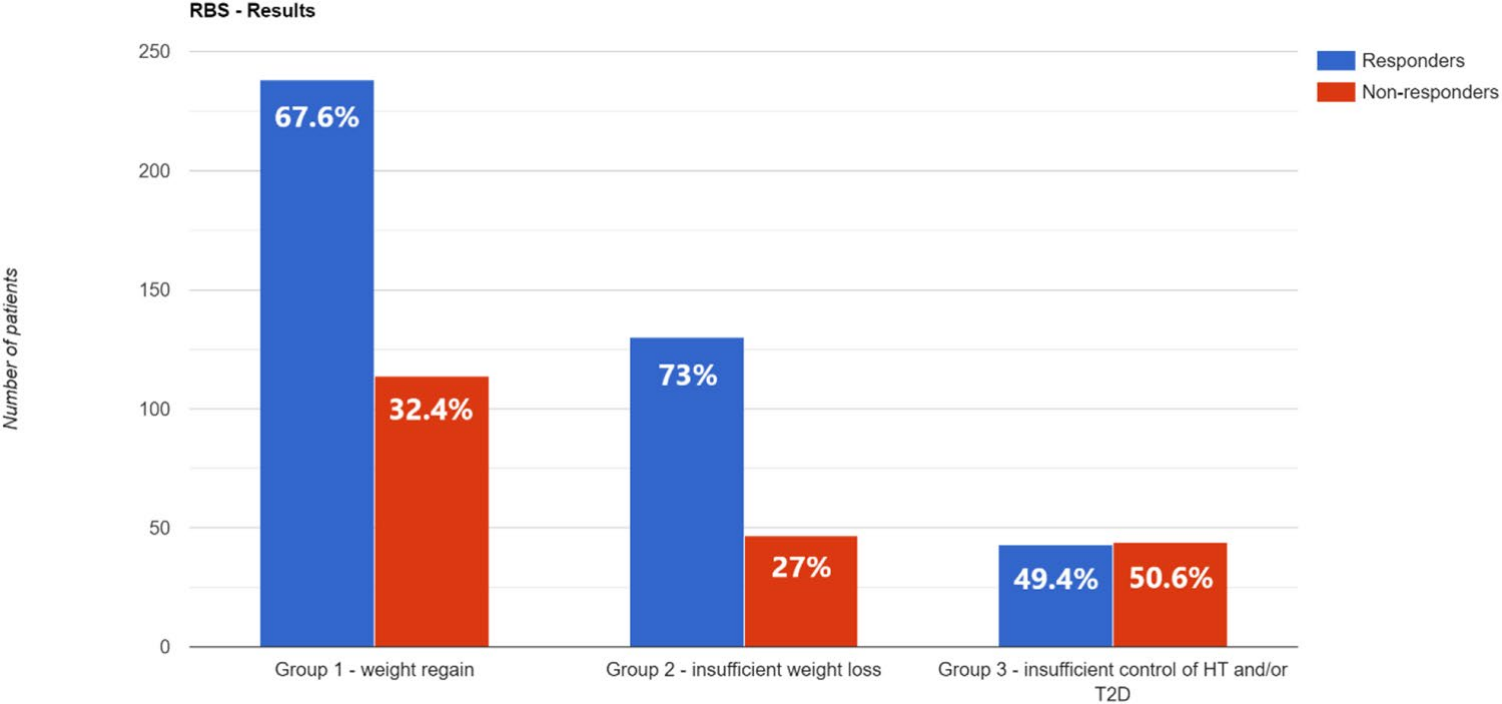
Graphic presentation of study groups’ response/non-response ratio

Figure 2 presents graphic presentation of study groups’ in terms of response and non-response ratios.

### Group 1: Patients with Weight Regain After Primary Procedure

In this study group, 238 of 352 patients (67.6%) exceeded 50% EWL after redo bariatric surgery. General characteristics are presented in Table 1. Patients from responsive and patients from non-responsive subgroups did not statistically differ in sex, age, maximal BMI, BMI before primary procedure, duration of obesity, smoking, alcohol consumption, nonsteroidal anti-inflammatory drug (NSAID), or anticoagulation intake. Associated medical *conditions* such as hypertension, type 2 diabetes mellitus, asthma, obstructive sleep apnea, and chronic obstructive pulmonary disease also did not differ both subgroups. Active smokers did not undergo bariatric surgery—all smoking patients included in the study were advised to quit smoking tobacco at least 6 weeks before the surgery and to continue not smoking after the surgery. Prior gastric balloon treatment and type of primary procedure were not contributing factor in achieving satisfying weight loss after redo-surgery, as well as time interval between primary and redo-surgery.

**Table 1:** General characteristics with primary bariatric treatment and qualification for RBS details: group 1 (patients with weight regain after primary procedure)

	Group 1: non-responders (< 50% EWL)	Group 1: responders (≥ 50% EWL)	*p* value
n (%)	114 (32.4%)	238 (67.6%)	n/a
Male/female, *n* (%)	33/81 (28.6%/71.5%)	52/186 (21.9%/78.2%)	0.145
Median age, years (IQR)	36 (31–43)	38 (31–44)	0.163
Median maximal BMI, kg/m^2^ (IQR)	47.2 (42.5–52.1)	46.5 (42.2–52.6)	0.444
Median BMI before primary procedure, kg/m^2^ (IQR)	46.2 (41.5–50.5)	45.0 (40.5–50.2)	0.335
Duration of obesity, n (%)			
< 5 years	3 (2.6%)	10 (4.2%)	0.828
5–15 years	55 (48.2%)	110 (46.2%)	
> 15 years	56 (49.2%)	118 (49.6%)	
Smoking, *n* (%)	14 (12.3%)	29 (12.2%)	0.896
Alcohol consumption, *n* (%)	31 (27.2%)	70 (29.4%)	0.696
NSAID or anticoagulation > once a week, *n* (%)	12 (10.5%)	18 (7.6%)	0.383
Type 2 diabetes mellitus, *n* (%)	19 (16.7%)	50 (21.0%)	0.337
Hypertension, *n* (%)	43 (37.7%)	87 (36.6%)	0.832
Asthma, obstructive sleep apnea, chronic obstructive pulmonary disease, *n* (%)	6 (5.3%)	14 (5.9%)	0.814
Prior gastric balloon treatment, *n* (%)	6 (5.3%)	22 (9.24%)	0.280
Type of primary procedure, *n* (%)			0.125
LSG	65 (57.0%)	152 (63.9%)	
RYGB	3 (2.6%)	1 (0.4%)	
VBG	9 (7.9%)	18 (7.6%)	
OAGB	1 (0.9%)	0	
AGB	36 (31.6%)	63 (26.5%)	
GP	0	4 (1.7%)	
Median lowest BMI after primary procedure, kg/m^2^ (IQR)	33.8 (28.9–38.5)	30.8 (27.5–35.6)	** < 0.001**
Median interval between primary procedure and RBS, years (IQR)	5 (3–8)	5 (3–7)	0.528
Remission of type 2 diabetes mellitus, *n* (%)	3 (15.8%)	14 (28.0%)	0.359
Remission of hypertension, *n* (%)	6 (14.0%)	12 (13.0%)	0.899
Treatment continued in center that performed primary procedure, *n* (%)	62 (54.4%)	156 (66.1%)	**0.034**
BMI pre-RBS, kg/m^2^ (IQR)	43.1 (39.7–48.3)	39.1 (34.6–43.5)	** < 0.001**
Median difference in BMI pre-RBS and lowest after primary procedure, kg/m^2^ (IQR)	9.9 (6.0–14.1)	7.4 (4.3–10.8)	** < 0.001**
Median %TWL after PBS (with lowest weight achieved after PBS) (IQR)	29.9 (21.7 (36.5–15.4)	32.5 (27.2–40.6)	**0.002**
Median %TWL after PBS (with weight before RBS) (IQR)	6.4 (0.7–13.3)	15.7 (7.7–23.4)	** < 0.001**

Significant *p*-values (below 0.05) are bolded

*%EWL*, percentage of excess weight loss; *IQR*, inter-quartile range; *BMI*, body mass index; *NSAID*, non-steroid anti-inflammatory drugs; *LSG*, laparoscopic sleeve gastrectomy; *RYGB,* Roux-en-Y gastric bypass; *VBG*, vertical banded gastroplasty; *OAGB*, one anastomosis gastric bypass; *AGB*, adjustable gastric banding; *GP*, gastric plication; *RBS*, redo bariatric surgery; *%TWL*, percentage of total weight loss; *PBS*, primary bariatric surgery

Table 1 consists comparison of primary bariatric treatment between groups. Statistically significant differences were observed in median lowest BMI after primary procedure (lower in responsive group), treatment continued in center that performed primary procedure (more often observed in patients with better weight loss outcome), BMI at the moment of RBS (lower in responsive group), median difference in BMI between pre-RBS and lowest after primary procedure (lower in responsive group), median BMI after RBS (lower in responsive group), and remission of type 2 diabetes after RBS (corresponded with greater weight loss). Table 2 summarizes bariatric results in groups regarding RBS.

**Table 2:** RBS: group 1 (patients with weight regain after primary procedure)

	Group 1: non-responders (< 50% EWL)	Group 1: responders (≥ 50% EWL)	*p* value
*n* (%)	114 (32.4%)	238 (67.6%)	n/a
Median BMI after RBS, kg/m^2^ (IQR)	38.4 (34.7–41.9)	29.9 (27.4–33.0)	** < 0.001**
Type of RBS			0.510
LSG/re-SG	24 (21.1%)	46 (19.3%)	
Others (BPD-DS, SAGI, SASI)	2 (1.8%)	9 (3.8%)	
RYGB	49 (43.0%	89 (37.4%)	
OAGB	39 (34.2%)	94 (39.5%)	
Remission of type 2 diabetes mellitus, *n* (%)	1 (5.3%)	22 (44.0%)	** < 0.001**
Remission of hypertension, *n* (%)	8 (18.6%)	23 (26.4%)	0.280
Postoperative morbidity, *n* (%)	22 (19.3%)	52 (21.9%)	0.583
Median %TWL after RBS (IQR)	19.5 (12.9–24.6)	34.8 (30.8–41.2)	** < 0.001**

Significant *p*-values (below 0.05) are bolded

*%EWL*, percentage of excess weight loss; *RBS*, redo bariatric procedure; *LSG,* laparoscopic sleeve gastrectomy; *re-SG*, redo sleeve gastrectomy; *BPS-DS*, biliopancreatic diversion with duodenal switch; *SAGI*, single anastomosis gastro-ileal bypass; *SASI*, single anastomosis sleeve-ileal bypass; *RYGB*, Roux-en-Y gastric bypass; *OAGB*, one-anastomosis gastric bypass; *%TWL*, percentage total weight loss

All available risk factors were analyzed in univariate logistic regression models (supplementary material). Lowest BMI after primary procedure (*p* = 0.001), treatment continued in center that performed primary procedure (*p* = 0.035), BMI pre-RBS (*p* < 0.001), and difference in BMI pre-RBS and lowest after primary procedure (*p* = 0.001) were significantly increasing odds ratio of effective weight loss after RBS in univariate logistic regression models. Receiver operating characteristics (ROC) analysis was performed to find significant cut-off point for difference in BMI pre-RBS and lowest after primary procedure (AUC 0.62, 95% CI 0.55–0.68, *p* < 0.001). It was set at 10.6 kg/m^2^ and included in multivariate model. Increase by 10.6 kg/m^2^ in BMI from lowest after primary procedure to BMI measured before RBS was independently increasing odds ratio of unsatisfactory weight loss after RBS. ROC curve analysis sets cut-off point for difference in BMI pre-RBS and lowest after primary procedure at 10.6 (AUC 0.62, 95% CI 0.55–0.68, *p* < 0.001). Results of multivariate logistic regression are presented in Table 3.

**Table 3:** Multivariate logistic regression model: group 1 (patients with weight regain after primary procedure)

	OR	95% CI	*p* value
Treatment continued in center that performed primary procedure	1.27	0.78–2.06	0.330
Difference in BMI pre-RBS and lowest after primary procedure > 10.6	2.33	1.43–3.80	**0.001**

Significant *p*-values (below 0.05) are bolded

*OR*, odds ratio; *95% CI*, 95% confidence interval; *BMI*, body mass index; *RBS*, redo bariatric surgery

### Group 2: Patients with Insufficient Weight Loss After Primary Procedure

In this study group, 130 of 177 patients (73.4%) exceeded 50% EWL after redo bariatric surgery. General characteristics are presented in Table 4. Patients did not significantly differ in terms of sex, age, duration of obesity, smoking, alcohol consumption, NSAID or anticoagulation intake, type 2 diabetes, hypertension, and pulmonary conditions. Median maximal BMI was significantly greater in responsive group of patients as well as BMI before primary procedure. Prior gastric balloon treatment did not differ groups.

**Table 4:** General characteristics with primary bariatric treatment and qualification for RBS details: – group 2 (patients with insufficient weight loss after primary procedure)

	Group 2: non-responders (< 50% EWL)	Group 2: responders (≥ 50% EWL)	*p* value
*n* (%)	47 (37%)	130 (73%)	n/a
Male/female, *n* (%)	13/34 (28%/72%)	36/94 (28%/72%)	0.997
Median age, years (IQR)	38 (31–47)	40 (34–51)	0.053
Median maximal BMI, kg/m^2^ (IQR)	44.6 (40.5–50.0)	50.1 (44.9–54.5)	** < 0.001**
Median BMI before primary procedure, kg/m^2^ (IQR)	44.3 (40.2–47.4)	46.2 (42.2–50.4)	**0.026**
Duration of obesity, *n* (%)			
< 5 years	3 (6.4%)	6 (4.6%)	0.183
5–15 years	15 (31.9%)	62 (47.7%)	
> 15 years	29 (61.7%)	62 (47.7%)	
Smoking, *n* (%)	11 (23.4%)	16 (12.3%)	0.205
Alcohol consumption, *n* (%)	11 (23.4%)	43 (33.1%)	0.377
NSAID or anticoagulation > once a week, *n* (%)	6 (12.8%)	22 (16.9%)	0.858
Type 2 diabetes mellitus, *n* (%)	9 (19.2%)	44 (33.9%)	0.089
Hypertension, *n* (%)	24 (51.1%)	74 (56.9%)	0.489
Asthma, obstructive sleep apnea, chronic obstructive pulmonary disease, *n* (%)	3 (6.4%)	14 (10.8%)	0.382
Prior gastric balloon treatment, *n* (%)	3 (6.4%)	8 (6.2%)	0.598
Type of primary procedure, *n* (%)			**0.004**
LSG	24 (51.1%)	101 (77.7%)	
RYGB	2 (4.3%)	1 (0.8%)	
VBG	2 (4.3%)	1 (0.8%)	
OAGB	0	3 (2.3%)	
AGB	19 (40.4%)	23 (17.7%)	
GP	0	1 (0.8%)	
Median lowest BMI after primary procedure, kg/m^2^ (IQR)	38.7 (36.2–40.8)	37.1 (33.5–42.0)	0.260
Median interval between primary procedure and RBS, years (IQR)	4 (3–7)	2 (1–3)	** < 0.001**
Remission of type 2 diabetes mellitus, *n* (%)	0	11 (25.0%)	n/a
Remission of hypertension, *n* (%)	2 (8.3%)	13 (17.6%)	0.508
Treatment continued in center that performed primary procedure, *n* (%)	33 (70.2%)	110 (84.6%)	**0.032**
BMI pre-RBS, kg/m^2^ (IQR)	42.1 (37.8–45.0)	38.6 (34.3–42.5)	**0.002**
Median difference in BMI pre-RBS and lowest after primary procedure, kg/m^2^ (IQR)	0.8 (0–4.7)	0 (0–1.8)	**0.022**
Median %TWL after PBS (with lowest weight achieved after PBS) (IQR)	11.9 (9–24.4)	24.6 (17.4–30.8)	** < 0.001**
Median %TWL after PBS (with weight before RBS) (IQR)	6.8 (0.8–11.61)	21.7 (14.1–30.0)	** < 0.001**

Significant *p*-values (below 0.05) are bolded

*%EWL*, percentage of excess weight loss; *IQR*, inter-quartile range; *BMI*, body mass index; *NSAID*, non-steroid anti-inflammatory drugs; *LSG*, laparoscopic sleeve gastrectomy; *RYGB*, Roux-en-Y gastric bypass; *VBG*, vertical banded gastroplasty; *OAGB*, one anastomosis gastric bypass; *AGB*, adjustable gastric banding; *GP*, gastric plication; *RBS*, redo bariatric surgery; *%TWL*, percentage of total weight loss; *PBS*, primary bariatric surgery

Subgroups differed significantly in terms of: time interval between PBS and RBS (was two times longer in non-responsive group), continuity of treatment in the same center that performed PBS (higher in responsive group), BMI before RBS (lower in responsive group), median difference in BMI between pre-RBS and lowest after primary procedure (higher in non-responsive group), median BMI after RBS (lower in responsive group), and postoperative morbidity (more often observed in patients with > 50% EWL). Type of RBS performed was relevant to the outcome (*p* < 0.001) with the most common operation in responsive group being OAGB (34.0% vs 72.3%). Details of types of RBS are presented in Table 5. Remission of type 2 diabetes and hypertension after RBS corresponded with greater weight loss.

**Table 5:** RBS: group 2 (patients with insufficient weight loss after primary procedure)

	Group 2: non-responders (< 50% EWL)	Group 2: responders (≥ 50% EWL)	*p* value
*n* (%)	47	130	n/a
Median BMI after RBS, kg/m^2^ (IQR)	37.0 (34.1–41.3)	30.5 (27.8–33.9)	** < 0.001**
Type of RBS			** < 0.001**
LSG/re-SG	11 (23.4%)	13 (10.0%)	
Others (Fobi-pouch operation, gastric pouch reduction after RYGB, BPD-DS, SAGI)	3 (6.4%)	1 (0.8%)	
RYGB	17 (36.2%)	22 (16.9%)	
OAGB	16 (34.0%)	94 (72.3%)	
Remission of type 2 diabetes mellitus, *n* (%)	3 (33.3%)	25 (56.8%)	**0.021**
Remission of hypertension, *n* (%)	3 (12.5%)	27 (36.5%)	**0.037**
Postoperative morbidity, *n* (%)	2 (4.3%)	32 (24.6%)	**0.002**
Median %TWL after RBS (IQR)	18.2 (10.9–21.6)	38.7 (32.1–43.1)	** < 0.001**

Significant *p*-values (below 0.05) are bolded

*%EWL*, percentage of excess weight loss; *BMI*, body mass index; *RBS*, redo bariatric procedure; *LSG*, laparoscopic sleeve gastrectomy; *re-SG*, redo sleeve gastrectomy; *RYGB*, Roux-en-Y gastric bypass; *OAGB*, one-anastomosis gastric bypass; *BPS-DS*, biliopancreatic diversion with duodenal switch; *SAGI*, single anastomosis gastro-ileal bypass; *%TWL*, percentage total weight loss

All available risk factors were analyzed in univariate logistic regression models (supplementary material). Age (*p* = 0.037), maximal BMI (*p* = 0.001), not having AGB as primary bariatric procedure (*p* = 0.001), treatment continued in primary bariatric center (*p* = 0.035), median interval between primary procedure and RBS (*p* < 0.001), BMI pre-RBS (*p* = 0.008), difference in BMI pre-RBS and lowest after primary procedure (*p* = 0.002), and having OAGB as RBS (*p* = 0.001) were significantly increasing odds ratio of effective weight loss after RBS in univariate logistic regression models. Factors that were statistically significant were included in multivariate logistic regression model as shown in Table 6.

**Table 6:** Multivariate logistic regression analysis for factors contributing to bariatric success: group 2 (patients with insufficient weight loss after primary procedure)

	OR	95% CI	*p* value
Age	1.01	0.98–1.05	0.498
Maximal BMI	1.03	0.98–1.05	0.560
Primary bariatric procedure			
LSG	1.00		
RYGB	0.99	0.05–20.9	0.999
VBG	1.54	0.06–41.22	0.796
OAGB	n/a		
AGB	2.03	0.59–6.92	0.259
GP	n/a		
Treatment continued in primary bariatric center	0.66	0.20–2.13	0.486
Median interval between primary procedure and RBS	0.88	0.74–1.06	0.177
Difference in BMI pre-RBS and lowest after primary procedure	0.76	0.64–0.89	**0.001**
Types of RBS			
LSG/re-SG	1.00		
Others (Fobi-pouch operation, gastric pouch reduction after RYGB, BPD-DS, SAGI)	0.12	0.001–9.61	0.339
RYGB	0.76	0.20–2.86	0.686
OAGB	2.80	0.67–11.64	0.157

Significant *p*-values (below 0.05) are bolded

*OR*, odds ratio; *95% CI*, 95% confidence interval; *BMI*, body mass index; *LSG*, laparoscopic sleeve gastrectomy; *RYGB*, Roux-en-Y gastric bypass; *VBG*, vertical banded gastroplasty; *OAGB*, one anastomosis gastric bypass; *AGB*, adjustable gastric banding; *GP*, gastric plication; *RBS*, redo bariatric surgery; *LSG*, laparoscopic sleeve gastrectomy; *Re-SG*, redo sleeve gastrectomy; *BPD-DS*, biliopancreatic diversion with duodenal switch; *SAGI*, single anastomosis gastro-ileal bypass

In multivariate logistic regression analysis, difference in BMI pre-RBS and lowest after primary procedure occurred to be contributing factor to effective weight loss in this group of patients.

### Group 3: Patients with Insufficient Control of Obesity-Related Diseases After Primary Procedure

In this study group, 44 of 87 (50.6%) patients did not achieve remission of type 2 diabetes and/or hypertension after RBS and 43 of 87 (49.4%) did. General characteristics are presented in Table 7. Overall, there were 28 patients in remission of T2D and 26 patients in remission of NT. Patients did not significantly differ in terms of sex, age, median maximal BMI, median BMI before primary procedure, smoking, alcohol consumption, NSAID or anticoagulation intake, type 2 diabetes, hypertension, and pulmonary conditions. Duration of obesity was significantly longer in non-responsive group: 29 of 44 patients (65.9%) were obese for more than 15 years compared to 11 of 43 patients (25.6%) in responsive group. Prior gastric balloon treatment did not differ groups. There were no significant differences in types of primary bariatric procedures with LSG being the most common in both groups (Table 7).

**Table 7:** General characteristics with primary bariatric treatment and qualification for RBS details – group 3 (patients with insufficient control of obesity-related diseases after primary procedure)

	Group 3: non-responders	Group 3: responders (remission of HT and/or T2D)	*p* value
*n* (%)	44 (50.6%)	43 (49.4%)	n/a
Male/female, *n* (%)	17/27 (39%/61%)	11/32 (26%/74%)	0.193
Median age, years (IQR)	44 (36–53)	42 (35–50)	0.372
Median maximal BMI, kg/m^2^ (IQR)	51.3 (46.3–57.1)	51.2 (46.5–54.2)	0.586
Median BMI before primary procedure, kg/m^2^ (IQR)	48.3 (43.3–53.6)	47.0 (41.5–51.3)	0.174
Duration of obesity, *n* (%)			
< 5 years	3 (6.8%)	4 (9.3%)	**0.001**
5–15 years	12 (27.3%)	28 (65.1%)	
> 15 years	29 (65.9%)	11 (25.6%)	
Smoking, *n* (%)	3 (6.8%)	6 (14.0%)	0.245
Alcohol consumption, *n* (%)	17 (40.5%)	11 (30.6%)	0.363
NSAID or anticoagulation > once a week, *n* (%)	6 (13.6%)	10 (23.3%)	0.160
Type 2 diabetes mellitus, *n* (%)	25 (56.8%)	31 (72.1%)	0.137
Hypertension, *n* (%)	37 (84.1%)	36 (83.7%)	0.963
Asthma, obstructive sleep apnea, chronic obstructive pulmonary disease, *n* (%)	4 (9.1%)	7 (16.28%)	0.352
Prior gastric balloon treatment, *n* (%)	4 (9.1%)	5 (11.6%)	0.739
Type of primary procedure, *n* (%)			0.648
LSG	32 (72.7%)	35 (81.4%)	
RYGB	2 (4.6%)	1 (2.3%)	
VBG	2 (4.6%)	1 (2.3%)	
OAGB	0	1 (2.3%)	
AGB	8 (18.2%)	5 (11.6%)	
Median lowest BMI after primary procedure, kg/m^2^ (IQR)	37.5 (34.1–42.0)	34.9 (30.7–41.1)	0.066
Median interval between primary procedure and RBS, years (IQR)	4 (2.5–6)	3 (1–4)	**0.004**
Remission of type 2 diabetes mellitus, *n* (%)	1 (4.0%)	6 (19.4%)	0.117
Remission of hypertension, *n* (%)	0	5 (13.9%)	n/a
Treatment continued in center that performed primary procedure, n (%)	33 (75.0%)	30 (69.8%)	0.585
BMI pre-RBS, kg/m^2^ (IQR)	43.9 (39.5–49.0)	41.1 (34.4–45.3)	**0.011**
Median difference in BMI pre-RBS and lowest after primary procedure, kg/m^2^ (IQR)	6.0 (1.9–10.5)	1.3 (0–9.4)	0.052
Median %TWL after PBS (with lowest weight achieved after PBS) (IQR)	26.8 (18.9–33.1)	29.1 (21.3–38.2)	0.106
Median %TWL after PBS (with weight before RBS) (IQR)	12.0 (6.2–21.0)	20.9 (15.5–27.7)	**0.002**

Significant *p*-values (below 0.05) are bolded

*HT*, hypertension; *T2D*, type 2 diabetes; *IQR*, inter-quartile range; *BMI*, body mass index; *NSAID*, non-steroid anti-inflammatory drugs; *LSG*, laparoscopic sleeve gastrectomy; *RYGB*, Roux-en-Y gastric bypass; *VBG*, vertical banded gastroplasty; *OAGB*, one anastomosis gastric bypass; *AGB*, adjustable gastric banding; *RBS*, redo bariatric surgery; *%TWL*, percentage of total weight loss; *PBS*, primary bariatric surgery

Subgroups differed in median interval between primary procedure and RBS (longer in non-responsive group), BMI before RBS (higher in non-responsive group), and median BMI after RBS (higher in non-responsive group). Type of RBS was an important factor with OAGB being the most common procedure in both groups but definitely more often performed in responsive one (36.4% vs 76.7%); details are presented in Table 8.

**Table 8:** RBS: group 3 (patients with insufficient control of obesity-related diseases after primary procedure)

	Group 3: non-responders	Group 3: responders (remission of HT and/or T2D)	*p* value
n (%)	44 (50.6%)	43 (49.4%)	n/a
Median BMI after RBS, kg/m^2^ (IQR)	35.1 (32.3–40.4)	30.7 (27.2–37.1)	** < 0.001**
Type of RBS			**0.001**
LSG/re-SG	15 (34.1%)	3 (7.0%)	
Others (SASI, reduction of gastric pouch after RYGB)	3 (6.8%)	2 (4.7%)	
RYGB	10 (22.7%)	5 (11.6%)	
OAGB	16 (36.4%)	33 (76.7%)	
Remission of type 2 diabetes mellitus, *n* (%)	0	28 (90.3%)	** < 0.001**
Remission of hypertension, *n* (%)	0	26 (72.2%)	** < 0.001**
Postoperative morbidity, *n* (%)	9 (20.5%)	9 (20.9%)	0.956
Median %TWL after RBS (IQR)	29.2 (24.4–35.6)	39.1 (33.5–43.6)	** < 0.001**

Significant *p*-values (below 0.05) are bolded

*HT*, hypertension; *T2D*, type 2 diabetes; *BMI*, body mass index; *RBS*, redo bariatric procedure; *LSG*, laparoscopic sleeve gastrectomy; *re-SG*, redo sleeve gastrectomy; *SASI*, single anastomosis sleeve-ileal bypass; *RYGB*, Roux-en-Y gastric bypass; *OAGB*, one-anastomosis gastric bypass; *%TWL*, percentage of total weight loss

All available risk factors were analyzed in univariate logistic regression models (supplementary material). BMI before RBS (*p* = 0.020) and OAGB as the type RBS performed (*p* = 0.001) were significantly increasing odds ratio of response in this group. These factors were included in multivariate regression model as shown in Table 9.

**Table 9:** Multivariate logistic regression analysis for factors contributing to bariatric success: group 3 (patients with insufficient control of obesity-related diseases after primary procedure)

	OR	95% CI	*p* value
BMI pre-RBS > 45.5 kg/m^2^	0.48	0.15–1.49	0.201
Types of RBS			
LSG/re-SG	1.00		
Others (SASI, reduction of gastric pouch after RYGB)	2.81	0.31–25.78	0.362
RYGB	2.19	0.41–11.63	0.356
OAGB	7.23	1.67–31.33	**0.008**

Significant *p*-values (below 0.05) are bolded

*OR*, odds ratio; *95% CI*, 95% confidence interval; *BMI*, body mass index; *RBS*, redo bariatric surgery; *LSG*, laparoscopic sleeve gastrectomy; *Re-SG*, redo sleeve gastrectomy; *SASI*, single anastomosis sleeve-ileal bypass; *RYGB*, Roux-en-Y gastric bypass; *OAGB*, one anastomosis gastric bypass

ROC analysis was performed to find significant cut-off point for BMI before RBS (AUC 0.66 95% CI 0.54–0.77, *p* = 0.007). It was set at 45.5 kg/m^2^ and included in multivariate model along with types of performed RBS. OAGB as RBS was independently increasing odds ratio of response in complete remission of hypertension or/and type 2 diabetes (Table 10).

**Table 10:** Efficacy results’ comparison in relation to the type of redo-surgery in 3 groups

Group 1 (*n* = 352)		Group 2 (*n* = 177)		Group 3 (*n* = 87)	
Non-responders (*n* = 114)	Responders (*n* = 238)	Non-responders (*n* = 47)	Responders (*n* = 130)	Non-responders (*n* = 44)	Responders (*n* = 43)
LSG/re-SG (*n* = 70)		LSG/re-SG (*n* = 24)		LSG/re-SG (*n* = 18)	
24 (34.3%)	46 (65.7%)	11 (45.8%)	13 (54.2%)	15 (83.3%)	3 (16.7%)
RYGB (*n* = 138)		RYGB (*n* = 39)		RYGB (*n* = 15)	
49 (35.5%)	89 (64.5%)	17 (43.6%)	22 (56.4%)	10 (66.7%)	5 (33.3%)
OAGB (*n* = 133)		OAGB (*n* = 110)		OAGB (*n* = 49)	
39 (29.3%)	94 (70.7%)	16 (14.5%)	94 (85.5%)	16 (32.7%)	33 (67.3%)
Others (*n* = 11) (BPD-DS, SAGI, SASI)		Others (*n* = 4) (Fobi-pouch operation, gastric pouch reduction after RYGB, BPD-DS, SAGI)		Others (*n* = 5) (SASI, reduction of gastric pouch after RYGB)	
2 (18.2%)	9 (81.8%)	3 (75%)	1 (25%)	3 (60%)	2 (40%)

Definitions of response: > 50% EWL for groups 1 and 2, remission of T2D and/or HT for group 3

*RBS*, redo bariatric procedure; *LSG*, laparoscopic sleeve gastrectomy; *re-SG*, redo sleeve gastrectomy; *RYGB*, Roux-en-Y gastric bypass; *OAGB*, one-anastomosis gastric bypass; *BPD-DS*, biliopancreatic diversion with duodenal switch; *SAGI*, single anastomosis gastric-ileal bypass; *SASI*, single anastomosis sleeve-ileal bypass; *%EWL*, percentage of excess weight loss; *T2D*, type 2 diabetes; *HT*, hypertension

ROC curve analysis set cut-off point for BMI pre-RBS at 45.5 kg/m^2^ (AUC 0.66 95% CI 0.54–0.77, *p* = 0.007).

Complete results of univariate logistic regression models for all of study groups are presented in Tables 11–13 of Supplementary Material.

## Discussion

This publication is an attempt to analyze data from 12 Polish bariatric centers regarding redo bariatric surgery. In literature review, the data regarding risk factors for successful and unsuccessful RBS is scarce. We did not find another research that focused strictly on this topic. We did not find any agreed definition of success for RBS. Because indications for RBS vary, effectiveness of RBS should be considered regarding of its indications.

RBS is an approved method of treatment for obesity-associated medical *conditions*. Meta-analysis by Koh et al. demonstrated a 92% improvement and 50% remission of diabetes along with 81% improvement and 33% remission of hypertension. Additionally, they reported remission of hyperlipidemia in 37% and improvement of sleep apnea in 86% of patients [2]. RYGB seems to be more effective in improvement of diabetes than SG [16].

Band-related procedures (AGB, VGB) appear to require redo bariatric operations more often compared to other bariatric procedures, with RYGB being the most common redo bariatric surgery performed [2, 6, 17].

In the first consensus statement on redo bariatric surgery (2019), experts stated that it is not possible to lay down specific criteria for RBS in terms of BMI, weight loss, or weight regain after PBS. They agreed that decision to perform RBS should be individualized for every patient and made by multidisciplinary team. They stated that clinical response to PBS or RBS depends on a number of patient-related and procedure-related factors. They also agreed that it is undesirable to have strict definitions of “success” or “failure” after redo bariatric surgery, although this consensus required second round of voting [18].

According to the reviewed literature, main indications for RBS are inadequate weight loss, recurrence of obesity, and inadequate control of its associated *diseases* [5, 19]. Further, it includes GERD and other complications of primary procedures, the most common of which are those related to the gastric banding, but also marginal ulcers, anastomosis stricture, leak, nutritional deficiencies, and others.

Success of PBS in terms of weight loss is considered as a long-term loss of ≥ 50% EWL. While some authors point out that this is not optimal determinant of efficiency in treatment of clinically obesity, currently this seems to be valuable and simple indicator of success, including its recurrence. According to systematic review prepared by Mann et al., < 50% EWL at 18 months was the most frequent identified definition of lack of success [20].

This study has shown that patients with weight regain who were better responders for the primary procedure determined by lower BMI after surgery, less weight regain, and lower BMI at the time of RBS were more likely to be better responders after RBS. Specifically, in case of patients qualified for RBS because of weight regain, increase of BMI from lowest after primary surgery to the day of RBS < 10.6 kg/m^2^ increased odds for bariatric success 2.33 times.

The same definition of success was established for patients qualified for RBS because of insufficient weight loss. In this group, similar as in group 1, the difference in BMI between the time of RBS and lowest after primary procedure was independent factor of achieving success of RBS in multivariate logistic regression model. Higher maximal lifetime BMI and higher BMI before the primary procedure were also a predictor of good response in this group of patients.

This study also has shown that patients who underwent RBS because of insufficient control of obesity-related medical *conditions* were 7.23 times more likely to achieve remission of hypertension or type 2 diabetes when OAGB was performed as RBS (compared to LSG/re-SG). The superiority of OAGB over RYGB in this study derives from very long enzymatic loop length that were used. Common channel length is almost as short as in RYGB. OAGB was also associated with satisfying weight loss results: 70.7% patients from group 1 and 85.5% patients from group 2 achieved > 50% EWL after OAGB. OAGB as RBS was previously evaluated in other studies with satisfying results in terms of weight loss and control of associated *diseases*. In systematic review and meta-analysis by Kermansaravi et al., in patients who had OAGB as RBS, mean BMI loss was 15,16 kg/m^2^ (33.2% BMIL), remission of T2D was observed in 78.1% ± 14.2% patients and remission of HT in 74.7% ± 16.3% at 5-year follow up [21]. In another review by Parmar et al., patients who had OAGB as RBS after SG or AGB presented mean %EWL of 65.2% at 1 year and 68.5% at 2 years after redo-surgery [22].

## Conclusion

Redo bariatric surgery is an effective form of treatment in patients who did not achieve good results after primary bariatric operations. 67.6% of patients after PBS with weight regain (obesity recurrence) exceeded 50% EWL after RBS. In three-quarters of patients (73.7%) who did not achieve satisfactory weight loss after PBS to begin with, RBS was successful treatment leading to > 50% EWL. Half of all patients (49.4%) that underwent RBS because of insufficient control of hypertension and/or type 2 diabetes experienced remission of at least one of these conditions.

Greater BMI difference between the time of RBS and lowest after PBS was associated with smaller chance of success in patients that underwent RBS because of weight regain or insufficient weight loss. In group that underwent RBS because of insufficient control of obesity-related *diseases*, OAGB as RBS was associated with greater chance of success.

## Supplementary Information

Below is the link to the electronic supplementary material.

Supplementary file1 (DOCX 27 KB)

## References

[1] Ma P, Reddy S, Higa KD (2016). Revisional bariatric/metabolic surgery: what dictates its indications?. Curr Atheroscler Rep.

[2] Koh ZJ, Chew CAZ, Zhang JJY, Syn N, Kim G, Yan So JB, Shabbir A (2020). Metabolic outcomes after revisional bariatric surgery: a systematic review and meta-analysis. Surg Obes Relat Dis.

[3] Mirkin K, Alli VV, Rogers AM (2021). Revisional bariatric surgery. Surg Clin North Am.

[4] Małczak P, Pisarska-Adamczyk M, Zarzycki P, Wysocki M, Major P (2021). Hiatal Hernia Repair during laparoscopic sleeve gastrectomy: systematic review and meta-analysis on gastroesophageal reflux disease symptoms changes. Pol Przegl Chir Poland.

[5] Angrisani L, Santonicola A, Iovino P, Vitiello A, Higa K, Himpens J, Buchwald H, Scopinaro N (2018). IFSO Worldwide Survey 2016: Primary, endoluminal, and revisional procedures. Obes Surg.

[6] Clapp B, Harper B, Dodoo C, Klingsporn W, Barrientes A, Cutshall M, Tyroch A (2020). Trends in revisional bariatric surgery using the MBSAQIP database 2015–2017. Surg Obes Relat Dis.

[7] Migaczewski M, Czerwińska A, Rubinkiewicz M, et al. The prevalence of, and risk factors for, Barrett’s oesophagus after sleeve gastrectomy. Videosurgery Other Miniinvasive Tech [Internet]. 2021;16:710–4. Available from: 10.5114/wiitm.2021.107776. 10.5114/wiitm.2021.107776 PMC8669983 34950266

[8] Nurczyk K, Chan C-E, Skoczylas T, Wallner G (2022). Follow-up after bariatric surgery: are we effective enough?. Videosurgery and Other Miniinvasive Techniques.

[9] Major P, Wysocki M, Dworak J, et al. Are bariatric operations performed by residents safe and efficient? Surg Obes Relat Dis [Internet]. Elsevier; 2017;13:614–21. Available from: 10.1016/j.soard.2016.11.017. 10.1016/j.soard.2016.11.017 28159560

[10] Brethauer SA, Kothari S, Sudan R, et al. Systematic review on reoperative bariatric surgery: American Society for Metabolic and Bariatric Surgery revision task force. Surg Obes Relat Dis. 2014;10(5):952–72. 10.1016/j.soard.2014.02.014. 10.1016/j.soard.2014.02.014 24776071

[11] Riddle MC, Cefalu WT, Evans PH, et al. Consensus report: definition and interpretation of remission in type 2 diabetes. Diabetes Care. 2021;44(10):2438–44. 10.2337/dci21-0034. 10.2337/dci21-0034 PMC8929179 34462270

[12] Budzyński A, Major P, Głuszek S, et al. Polskie rekomendacje w zakresie chirurgii bariatrycznej i metabolicznej. Med Prakt – Chir. 2016;6:13–25.

[13] García-Caballero M, Carbajo M. One anastomosis gastric bypass: a simple, safe and efficient surgical procedure for treating morbid obesity. Nutr Hosp. 2004;19(6):372–5. 15672654

[14] Malczak P, Pisarska M, Piotr M, Wysocki MM, Budzynski A, Pedziwiatr M (2017). Enhanced recovery after bariatric surgery: systematic review and meta-analysis. Obes Surg United States.

[15] Sinha A, Jayaraman L, Punhani D, Chowbey P (2017). Enhanced recovery after bariatric surgery in the severely obese, morbidly obese, super-morbidly obese and super-super morbidly obese using evidence-based clinical pathways: a comparative study. Obes Surg.

[16] Yan J, Cohen R, Aminian A (2017). Reoperative bariatric surgery for treatment of type 2 diabetes mellitus. Surg Obes Relat Dis.

[17] Hjorth S, Näslund I, Andersson-Assarsson JC, et al. Reoperations after bariatric surgery in 26 years of follow-up of the Swedish obese subjects study. JAMA Surg. 2019;154(4):319–26. 10.1001/jamasurg.2018.5084 (Erratum in: JAMA Surg. 2019 Apr 1;154(4):368). 10.1001/jamasurg.2018.5084 PMC6484798 30601881

[18] Mahawar KK, Himpens JM, Shikora SA, et al. The first consensus statement on revisional bariatric surgery using a modified Delphi approach. Surg Endosc. 2020;34(4):1648–57. 10.1007/s00464-019-06937-1. 10.1007/s00464-019-06937-1 31218425

[19] Mahawar KK, Nimeri A, Adamo M, Borg CM, Singhal R, Khan O, Small PK (2018). Practices concerning revisional bariatric surgery: a survey of 460 surgeons. Obes Surg.

[20] Mann JP, Jakes AD, Hayden JD, Barth JH (2015). Systematic review of definitions of failure in revisional bariatric surgery. Obes Surg.

[21] Kermansaravi M, Shahmiri SS, DavarpanahJazi AH, et al. One anastomosis/mini-gastric bypass (OAGB/MGB) as revisional surgery following primary restrictive bariatric procedures: a systematic review and meta-analysis. Obes Surg. 2021;31(1):370–383. 10.1007/s11695-020-05079-x. 10.1007/s11695-020-05079-x PMC7809003 33118133

[22] Parmar CD, Gan J, Stier C, Dong Z, Chiappetta S, El-Kadre L, Bashah MM, Wang C, Sakran N (2020). One anastomosis/mini gastric bypass (OAGB-MGB) as revisional bariatric surgery after failed primary adjustable gastric band (LAGB) and sleeve gastrectomy (SG): a systematic review of 1075 patients. Int J Surg.

