# Supplementary material for: Analysis of the Factors Contributing to Bariatric Success After Laparoscopic Redo Bariatric Procedures: Results from Multicenter Polish Revision Obesity Surgery Study (PROSS)
Source: Obes Surg. 2022 Oct 15;32(12):3879–90. doi: 10.1007/s11695-022-06306-3 (PMC9672013; doi:10.1007/s11695-022-06306-3)
Supplement: Supplementary file 1 — Supplementary file1 (DOCX 27 KB) [file 11695_2022_6306_MOESM1_ESM.docx]

| Table 11. Univariate logistic regression analyses for factors contributing to bariatric success in Group 1 – patients with weight regain after primary procedure. | | | |
| --- | --- | --- | --- |
|  | OR | 95%CI | p-value |
| Female | 1.46 | 0.88-2.42 | 0.147 |
| Age | 1.00 | 0.99-1.02 | 0.846 |
| Maximal BMI | 0.99 | 0.96-1.01 | 0.367 |
| Median BMI before primary procedure | 0.99 | 0.96-1.01 | 0.243 |
| Duration of obesity |  |  |  |
| <5 years | 1.00 |  |  |
| 5-15 years | 0.66 | 0.17-2.55 | 0.546 |
| >15 years | 0.69 | 0.18-2.67 | 0.595 |
| Smoking | 0.95 | 0.46-1.97 | 0.896 |
| Alcohol consumption | 1.12 | 0.64-1.95 | 0.696 |
| NSAID or anticoagulation > once a week | 0.69 | 0.30-1.60 | 0.385 |
| Type 2 diabetes mellitus | 1.33 | 0.74-2.38 | 0.338 |
| Hypertension | 0.95 | 0.60-1.51 | 0.832 |
| Asthma, obstructive sleep apnea, chronic obstructive pulmonary disease | 1.13 | 0.42-3.01 | 0.814 |
| Prior gastric balloon treatment | 1.83 | 0.72-4.67 | 0.202 |
| Types of primary procedure |  |  |  |
| LSG | 1.00 |  |  |
| RYGB | 0.14 | 0.02-1.40 | 0.094 |
| VBG | 0.86 | 0.37-2.00 | 0.719 |
| OAGB | n/a |  |  |
| AGB | 0.75 | 0.45-1.24 | 0.258 |
| GP | n/a |  |  |
| Lowest BMI after primary procedure | 0.94 | 0.91-0.98 | **0.001** |
| Interval between primary procedure and RBS | 1.01 | 0.99-1.03 | 0.372 |
| Treatment continued in center that performed primary procedure | 1.64 | 1.04-2.56 | **0.035** |
| BMI pre-RBS | 0.91 | 0.88-0.94 | **<0.001** |
| Difference in BMI pre-RBS and lowest after primary procedure | 0.93 | 0.90-0.97 | **0.001** |
| Types of RBS |  |  |  |
| re-SG | 1.00 |  |  |
| Others (BPD-DS, SAGI, SASI) | 2.35 | 0.47-11.74 | 0.299 |
| OAGB | 0.95 | 0.52-1.73 | 0.862 |
| RYGB | 1.26 | 0.68-2.34 | 0.468 |

OR – odds ratio

95%CI – 95% confidence interval

BMI – body mass index

NSAID – non-steroid anti-inflammatory drugs

LSG – laparoscopic sleeve gastrectomy

RYGB – Roux-en-Y gastric bypass

VGB – vertical gastric banding

OAGB – one-anastomosis gastric bypass

AGB – adjustable gastric banding

GP – gastric plication

RBS – redo bariatric surgery

Re-SG – redo sleeve gastrectomy

BPD-DS – biliopancreatic diversion with duodenal switch

SAGI – single anastomosis gastric-ileal bypass

SASI – single anastomosis sleeve-ileal bypass

| Table 12. Univariate logistic regression analyses for factors contributing to bariatric success in Group 2 – patients with insufficient weight loss after primary procedure. | | | |
| --- | --- | --- | --- |
|  | OR | 95%CI | p-value |
| Female | 0.99 | 0.47-2.10 | 0.997 |
| Age | 1.03 | 0.00-.107 | **0.037** |
| Maximal BMI | 1.10 | 1.04-1.67 | **0.001** |
| Median BMI before primary procedure | 1.05 | 0.99-1.11 | 0.056 |
| Duration of obesity |  |  |  |
| <5 years | 1.00 |  |  |
| 5-15 years | 2.36 | 0.50-11.07 | 0.277 |
| >15 years | 1.22 | 0.27-5.50 | 0.794 |
| Smoking | 0.48 | 0.18-1.24 | 0.130 |
| Alcohol consumption | 1.69 | 0.66-4.31 | 0.273 |
| NSAID or anticoagulation > once a week | 1.32 | 0.41-4.23 | 0.646 |
| Type 2 diabetes mellitus | 2.16 | 0.96-4.87 | 0.063 |
| Hypertension | 1.27 | 0.65-2.47 | 0.489 |
| Asthma, obstructive sleep apnea, chronic obstructive pulmonary disease | 1.77 | 0.49-6.46 | 0.387 |
| Prior gastric balloon treatment | 0.96 | 0.24-3.79 | 0.956 |
| Primary bariatric procedure |  |  |  |
| LSG | 1.00 |  |  |
| RYGB | 0.12 | 0.01-1.37 | 0.087 |
| VBG | 0.12 | 0.01-1.37 | 0.087 |
| OAGB | n/a |  |  |
| AGB | 0.29 | 0.14-0.61 | **0.001** |
| GP | n/a |  |  |
| Lowest BMI after primary procedure | 0.98 | 0.93-1.03 | 0.387 |
| Treatment continued in primary bariatric center | 2.33 | 1.06-5.12 | **0.035** |
| Median interval between primary procedure and RBS | 0.77 | 0.68-0.87 | **<0.001** |
| BMI pre-RBS | 0.93 | 0.88-0.98 | **0.008** |
| Difference in BMI pre-RBS and lowest after primary procedure | 0.87 | 0.80-0.95 | **0.002** |
| Types of RBS |  |  |  |
| LSG/re-SG | 1.00 |  |  |
| Others (Fobi-pouch operation, gastric pouch reduction after RYGB, BPD-DS, SAGI) | 0.28 | 0.03-3.11 | 0.302 |
| RYGB | 1.10 | 0.39-3.04 | 0.862 |
| OAGB | 4.97 | 1.90-13.01 | **0.001** |

OR – odds ratio

95%CI – 95% confidence interval

BMI – body mass index

NSAID – non-steroid anti-inflammatory drugs

LSG – laparoscopic sleeve gastrectomy

RYGB – Roux-en-Y gastric bypass

VGB – vertical gastric banding

OAGB – one-anastomosis gastric bypass

AGB – adjustable gastric banding

GP – gastric plication

RBS – redo bariatric surgery

Re-SG – redo sleeve gastrectomy

BPD-DS – biliopancreatic diversion with duodenal switch

SAGI – single anastomosis gastric-ileal bypass

| Table 13. Univariate logistic regression analyses for factors contributing to bariatric success in Group 3 – patients with insufficient control of comorbidities after primary procedure. | | | |
| --- | --- | --- | --- |
|  | OR | 95%CI | p-value |
| Female | 1.83 | 0.73-4.57 | 0.195 |
| Age | 0.98 | 0.95-1.01 | 0.126 |
| Maximal BMI | 0.99 | 0.94-1.05 | 0.799 |
| Median BMI before primary procedure | 0.97 | 0.92-1.02 | 0.245 |
| Duration of obesity |  |  |  |
| <5 years | 1.00 |  |  |
| 5-15 years | 1.84 | 0.35-9.61 | 0.469 |
| >15 years | 0.30 | 0.06-1.59 | 0.157 |
| Smoking | 4.41 | 0.11-17.38 | 0.341 |
| Alcohol consumption | 0.65 | 0.25-1.66 | 0.364 |
| NSAID or anticoagulation > once a week | 2.33 | 0.70-7.77 | 0.167 |
| Type 2 diabetes mellitus | 1.96 | 0.80-4.80 | 0.139 |
| Hypertension | 0.97 | 0.31-3.05 | 0.963 |
| Asthma, obstructive sleep apnea, chronic obstructive pulmonary disease | 1.94 | 0.53-7.20 | 0.319 |
| Prior gastric balloon treatment | 1.32 | 0.33-5.27 | 0.698 |
| Primary bariatric procedure |  |  |  |
| LSG | 1.00 |  |  |
| RYGB | 0.46 | 0.04-5.29 | 0.531 |
| VBG | 0.46 | 0.04-5.29 | 0.531 |
| OAGB | n/a |  |  |
| AGB | 0.57 | 0.17-1.93 | 0.367 |
| GP | n/a |  |  |
| Lowest BMI after primary procedure | 0.94 | 0.88-1.01 | 0.090 |
| Treatment continued in primary bariatric center | 0.77 | 0.30-1.98 | 0.586 |
| Median interval between primary procedure and RBS | 0.87 | 0.76-1.01 | 0.064 |
| BMI pre-RBS | 0.93 | 0.88-0.99 | **0.020** |
| Difference in BMI pre-RBS and lowest after primary procedure | 0.96 | 0.89-1.03 | 0.243 |
| Types of RBS |  |  |  |
| LSG/re-SG | 1.00 |  |  |
| Others (SASI, reduction of gastric pouch after RYGB) | 3.33 | 0.38-23.39 | 0.278 |
| RYGB | 2.50 | 0.49-12.89 | 0.273 |
| OAGB | 10.31 | 2.61-40.82 | **0.001** |

OR – odds ratio

95%CI – 95% confidence interval

BMI – body mass index

NSAID – non-steroid anti-inflammatory drugs

LSG – laparoscopic sleeve gastrectomy

RYGB – Roux-en-Y gastric bypass

VGB – vertical gastric banding

OAGB – one-anastomosis gastric bypass

AGB – adjustable gastric banding

GP – gastric plication

RBS – redo bariatric surgery

Re-SG – redo sleeve gastrectomy

SASI – single anastomosis sleeve-ileal bypass

List of centers that participated in the study:

1. 2nd Department of General Surgery, Jagiellonian University Medical College, Krakow, Poland

2. Department of General Surgery and Surgical Oncology, Ludwik Rydygier Memorial Hospital, Krakow, Poland

3. Department of General and Endoscopic Surgery, EuroMediCare Specialist Hospital and Clinic, Wroclaw, Poland

4. Department of General and Endocrine Surgery, Medical University of Bialystok, Bialystok, Poland

5. Department of General, Endocrine and Transplant Surgery, Medical University of Gdansk, Gdansk, Poland

6. Surgery Clinic Mazan, Katowice, Poland

7. Department of General and Oncological Surgery, Ceynowa Hospital, Wejherowo, Poland

8. Department of General, Gastroenterological, and Oncological Surgery, Collegium Medicum Nicolaus Copernicus University, Torun, Poland

9. Department of General, Oncological and Digestive Tract Surgery, Centre of Postgraduate Medical Education, Orłowski Hospital, Warsaw, Poland

10. Department of General, Minimally Invasive and Elderly Surgery, University of Warmia and Mazury, Olsztyn, Poland

11. Department of General, Oncological, Metabolic and Thoracic Surgery, Military Institute of Medicine, Warsaw, Poland

12. Department of General and Vascular Surgery, Polanica Zdroj, Poland
